# Involving patients in healthcare research is well documented but can it work in lab-based research?

**DOI:** 10.1186/s40900-023-00500-y

**Published:** 2023-10-12

**Authors:** Adele E. Connor, Claire Hughes, Lea Schäfer, Lorraine McNally, Deirdre O’ Raw, Katayoun Bahramian, Bridget Carr, Ingrid Halligan Dunne, Joanne Lysaght, Sharon A. O’ Toole, Jeremy C. Simpson, Antoinette S. Perry

**Affiliations:** 1 School of Biology and Environmental Science, University College Dublin, Dublin, Ireland; 2 Cancer Biology and Therapeutics Laboratory, Conway Institute of Biomolecular and Biomedical Research, University College Dublin, Dublin, Ireland; 3 Cell Screening Laboratory, University College Dublin, Dublin, Ireland; 4 OvaCare, Cork, Ireland; 5 Cancer Immunology and Immunotherapy Group, Department of Surgery, Trinity Translational Medicine Institute, Trinity College Dublin, Dublin, Ireland; 6 Department Obstetrics and Gynaecology, Trinity College Dublin, Dublin, Ireland; 7 OvaCare Board and Medical Panel, Cork, Ireland; 8 Conway Institute of Biomolecular and Biomedical Research, University College Dublin, Dublin, Ireland

**Keywords:** PPI, Ovarian cancer, Precision medicine, Translational research

## Abstract

Public and Patient Involvement in research is becoming a requirement on most research funding applications; this includes both healthcare and lab-based research. Whilst case studies and practical guides have been developed and are well documented for PPI in healthcare research, there is very little guidance available for PPI in lab-based research. In this piece we discuss our experience of how we have successfully involved patients in our translational cancer research, which is focused on developing personalised treatment for high-grade serous ovarian cancer. We discuss the benefits it has made to both our research and to us as researchers. The patients involved write about their experience, what they enjoyed, and the benefits they felt. Although PPI is quite topical and is being widely discussed, there is hesitancy among researchers, especially those in lab-based research about getting started because of a lack of practical guidance about how to implement it. Here, we have shared our experience, hopefully providing a practical example of how PPI can be incorporated into a lab-based research project.

## Abstract (plain language summary)

This piece is co-authored by researchers and ovarian cancer patients and presents their experience of patient involvement in a laboratory-based cancer research project focused on the personalised treatment of high-grade serous ovarian cancer. Discussions with five ovarian cancer patients about their treatment experience highlighted the fact that drugs showing equivalent clinical efficacy are not necessarily tolerated equally by individual patients. This led researchers to alter their original experimental design, by including a number of the same drug type instead of focusing on only one. The researchers also discuss the benefits it has made to both the research and to them as researchers. The patients involved write about their experience, what they enjoyed, and the benefits they felt.

## What is PPI?

Public and Patient Involvement (PPI) is quickly becoming a requirement for most research funding applications, but what exactly is it? How do we do it? And why is it important?

Scientists often formulate research questions around perceived unmet needs of patients. But how can we truly appreciate these needs without ever having spoken to patients? PPI is defined by the National Institute for Health and Care Research as ‘…research being carried out ‘with’ or ‘by’ members of the public rather than ‘to’, ‘about’ or ‘for’ them [1].’ This means establishing good relationships between researchers and patients. For example, discussing and designing research questions *together*, so that the lived experiences of patients can inform and guide investigations, and sharing research updates so the project remains patient focused throughout. Scientists and patients want the same thing, to reduce disease burden and improve outcomes, so it is imperative to work together to find the best solutions.

PPI has benefited healthcare research in numerous ways, such as improved patient participation by making language more understandable [2], and better community engagement when patients were involved in dissemination [3]. Benefits felt by patients include self-confidence and empowerment [4], an opportunity to contribute and provide valued information [4], and having something positive come from their illness [4, 5]. Although some resources exist for involvement in lab-based research [6] it isn’t as well documented and so it can feel more difficult to start. In particular we have found that there is often a misunderstanding by lab-based researchers when they are first introduced to PPI. Many think PPI involves patients running experiments in the lab, which obviously is not the case. However, the experimental process can be informed and improved by talking and discussing the lived experience with patients. Herein, we share the patient and researcher perspective, highlighting how the experience is mutually beneficial and important in shaping laboratory research. Patients who get involved with research projects act as advocates for this research and this can be mutually beneficial.

## Establishing PPI relationships

A group of five ovarian cancer patients were invited to a one day workshop at our cancer research laboratory. Two of the participants were already members of our patient advisory committee, while the other three were new to the project.

**Fig. 1:**
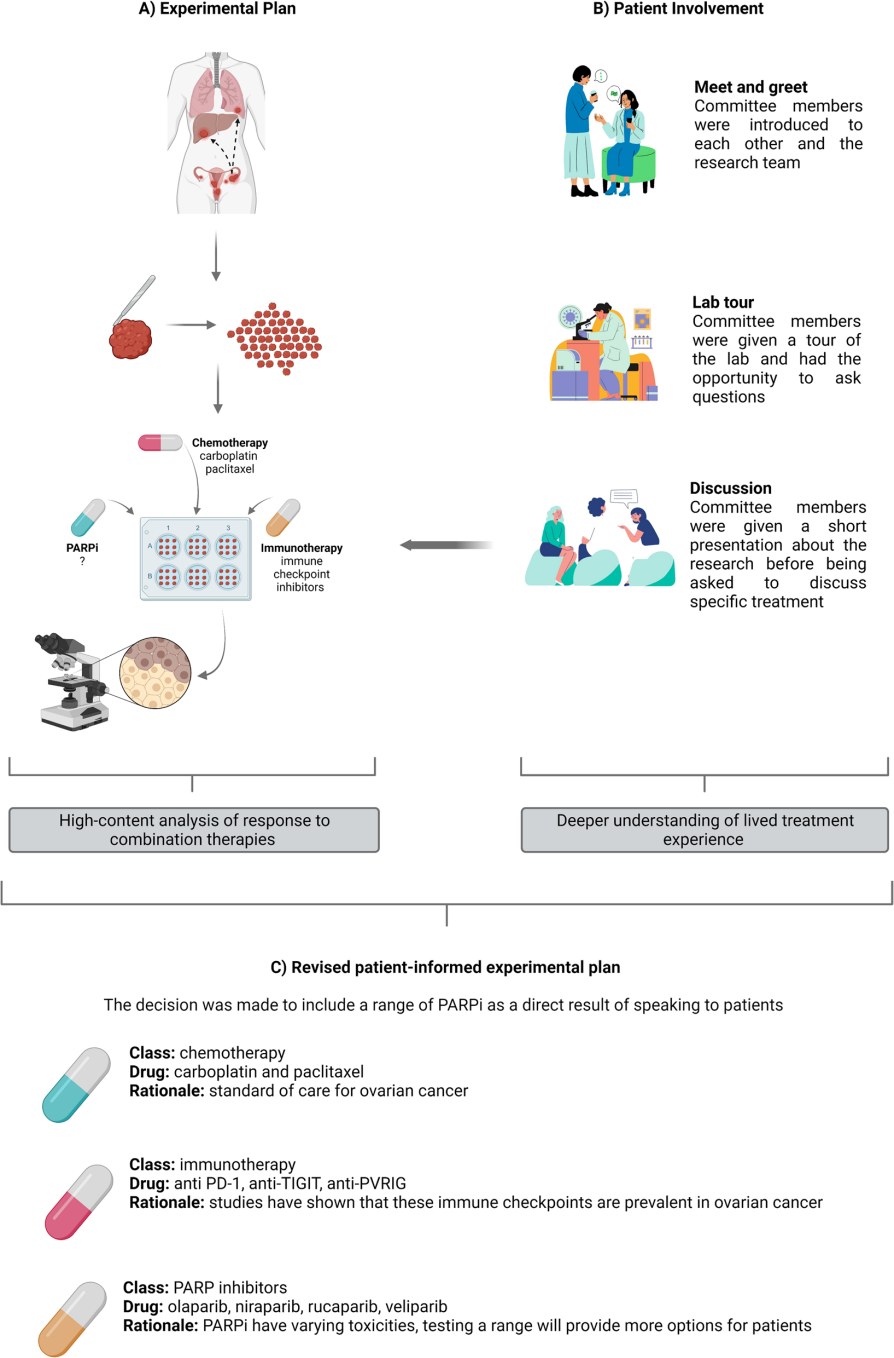
Involving patients in lab-based translational research. **A** The experimental plan. Patients underogoing surgery as part of their treatment plan can choose to donate their tumours to research. There tumours are dissected into ‘mini tumours’ which are then treated with different drug combinations in the lab and assessed via microscopy. **B** Patient involvement. Patient committee memebrs had a meet and greet, followed by a lab tour and then finally a discussion about treatment experience. This discussion fed directly into the experimental plans. The decision to include a range of PARPi in our experiments, instead of selecting just one, came as a direct result of speaking to patients

The workshop involved an initial meet and greet where committee members were introduced to each other and the research team. This was followed by a lab tour and a group discussion, where committee members watched a short presentation about the research project and were then asked specific questions about their treatment experience (Fig. 1). The experience of the researcher and the patient participants are discussed in the below sections.

## Early stage researcher perspective: Adele Connor, Ph.D. student

“As a Ph.D. researcher focused on ovarian cancer, my relationship with patients began when I was applying for my Ph.D. scholarship. I reached out to OvaCare, an Irish ovarian cancer support network [7] and was invited to their coffee morning where I presented my research and plans for patient involvement. This led to the formation of my patient advisory committee, and together we developed a terms of reference document. This document defined the purpose of our patient advisory committee and specified what is expected of both patient participants and researchers in terms of time commitment and discussion topics. We had our first meeting at the end of my first year, where each woman shared their story. My research focuses on imaging tumour cell responses to novel drug combinations and so one question I needed to answer was what drug(s) I should be testing (Fig. 1). Like all lab-based research, my project has a finite duration and budget, so I must be selective in choosing which drugs to take forward. Clinical trials are an important source of safety and efficacy data [8]. However, I lacked perspective on how tolerable these drugs are for patients or what quality of life is like living on them. This is where patient involvement was critical. I listened to people with first-hand experience, the experts on cancer treatment: the patients.

I learned that PARP inhibitors (PARPi) the ‘…breakthrough story for ovarian cancer over the past decade’ [9] are not without side effects. Discussions with patients about their treatment experience highlighted the fact that drugs showing equivalent clinical efficacy are not necessarily tolerated equally by individual patients [10]. This led me to alter my original experimental design (from testing Olaparib only), instead broadening my drug testing regimen to include multiple PARPi that have shown promising results in clinical trials (Niraparib, Rucaparib, Veliparib) (Fig. 1). So, involving patients in my research educated me on the need to factor in clinical efficacy as well as patient experiences when selecting drug compounds for evaluation on my patient-derived tumour models.

Completing a PhD can be really gruelling; the early mornings, late nights, failed experiments, mountains of papers to read, hours of laboratory demonstrating. It can be easy to forget why you’re doing it all in the first place. Speaking to patients and spending time with them re-energises me to keep moving forward.”

## Senior researcher perspective: Antoinette Perry, Associate Professor of Cell and Molecular Biology

“I have spent my career in cancer research, the last 25 years. Over this time, I have had the opportunity to work with and be mentored by wonderful scientists and clinicians in Ireland and abroad. I have travelled to centres of excellence to collaborate, upskill and broaden my perspectives. However, my experience involving patients in my research only began when I took up a faculty position in University College Dublin in 2015. Initiatives such as “The Patient Voice in Cancer Research” [11] really opened my eyes to the value that collaborating with patients could bring to our research programme. Adele was the first one of my students to establish a patient advisory committee, and I was amazed to see how much the women brought to the project, even from the earliest phases of project conception and funding application. The generosity, honesty, openness, bravery and dedication of our patients is truly humbling and makes me feel honoured that we could make a small contribution to the field”.

## Patients’ perspectives

A group of five patient participants were invited to University College Dublin where they were first given a tour of the laboratories. They were able to view ovarian cancer cells under a microscope and had the opportunity to ask any and all questions. This was followed up by a brief presentation about the research where the patients were given four specific questions about their treatment experience, which helped to guide a discussion. This was held in a private meeting room over tea and biscuits. The day was rounded off by a lunch at the University club restaurant, where everyone could get to know each other a little better, outside the confines of ‘patient’ and ‘researcher’.

Below are, in their own words, how the patient participants felt about the day.

## Patient participant perspective: Lorraine McNally

“I was very nervous meeting the other ladies. Adele made me feel very welcome and gave us a great tour of the labs and showed us cancer cells. What amazed me most was each one of us had a different route to being diagnosed with ovarian cancer. I felt so safe to be with people who understand what I am going through. We all understood each other’s struggles. I really enjoyed the day and I’m happy to know there is work going on in labs trying to find a cure for this awful cancer”.

## Patient participant perspective: Deirdre O’ Raw

“When I first signed up for this meeting, I was unsure of exactly what would happen. When we got to the lab, we got to see exactly how testing was done on specimens, this was very interesting as I like to know how things work and it was great to learn about how the different methods of treatment are tested. We also discussed our different treatments, it is strange to think that although we all have ovarian cancer that all of our treatments were very different. It was interesting to learn about where all the other women are with their disease, seeing people who were five years into their post treatment routine was a double-edged sword for me: there was only one of the women at that five-year point who had no tumours, this was quite frightening, although it was good to know that the other women were living with tumours. The discussion really brought home the reality of ovarian cancer and the fact that I would always be waiting for the next occurrence. The lunch was the perfect opportunity to have a more informal talk about everything and there were some humorous stories about everything we had all been through. The thing that really sticks with me from the day is that there is so much new research being done into ovarian cancer”.

## Patient participant perspective: Katayoun Bahramian

“Although I’ve worked on campus for 4 years, I had never stepped foot into a lab until we gathered for the ovarian cancer advisory committee meeting. It was fascinating to hear from Adele about her work and to see the specialized equipment. She explained complex biological concepts in an easy-to-understand manner and answered any questions we had. I am fortunate that my gynae cancers were detected at an early stage, which meant that my treatment plan was relatively straightforward. As a result, I felt guilty sharing that information among women whose experience of cancer was and is more complicated. Nevertheless, it was wonderful to meet and connect with others diagnosed with ovarian cancer. The discussion that we had over lunch was just as valuable. No better way to build trust than to break bread together and share a cuppa. I feel immense gratitude for the researchers’ sincere interest in capturing our voices and experiences.”

## Patient participant perspective: Ingrid Halligan Dunne

“The meeting was a breath of fresh air! The research team took the time to chat with us and explain their research into ovarian cancer. We each got to explain the treatment plan we were on and how it was working for us. The lab tour was a real treat and certainly gave us so much hope for women being diagnosed in the future. Adele really has a wonderful way of imparting her knowledge so that we understood how each step works. While I did not know what to expect on the day, I was delighted I went along, even to feel that I had helped in some way. Also there is no better tonic than meeting people that have had a similar diagnosis. The informal chat at lunch with the researchers and fellow patients was invaluable, it was truly great to see ovarian cancer had not taken away our sense of humour”.

## Patient participant perspective: Bridget Carr

“When it was first suggested we visit UCD, I didn't know what to expect. I was nervously excited to see the workings of the lab and to meet fellow ovarian cancer patients. It was fascinating to see all the equipment and see how research is carried out in a lab. Everything was explained in simple terminology and all our questions were answered. Following the lab tour we had a meeting. It was comforting to listen to the other ladies talk about their experiences and to know you are not alone. What struck me most was the genuine interest from Adele about our lived experience. She listened, took notes and asked many questions. It made me feel like my contribution was valued and important.

Adele truly understands the concept of "nothing about us without us". I look forward to our next meeting in UCD and being part of this very important research for ovarian cancer”.

The key benefits of public and patient participation in research are summarised in Table 1, from both the researcher and patient perspective.

**Table 1:** Involving patients in research is mutually beneficial for both scientists and patients

Benefits of patient involvement in research	
Benefits for researchers	Benefits for patients
Re-energised motivation for your work with a reminder of why we do this research	Discussing their experience with people who understand exactly what they are going through; if working with a group of participants
Research is focused on the unmet need(s) of patients	The knowledge that work is underway to tackle this cancer
A greater understanding of the lived experience	Opportunity to understand how cancer research works and how treatments are tested
Questions you may never have considered or thought of can be incorporated	Opportunity to ask questions about cancer research
	Thankful for researchers sincere interest in capturing patients voices

## Progressing the PPI relationship

Following on from this one day laboratory tour, workshop, and lunch the three participants who were new to the project decided to join our patient advisory committee. The contributions of our patient advisory committee are immensely valuable. In addition to discussions around research goals and priorities in other ovarian cancer research projects in our lab, notable examples include:

- Providing feedback on a lay oral presentation given by the early stage researcher at a national scientific conference, the Irish Association for Cancer Research [12].
- Co-applicants on a successful application to the Irish Cancer Society for a “Public and Patient Involvement Award 2022”. This project entitled ‘Broadening Patient Involvement in Ovarian Cancer Research’ began in March 2023 and has already resulted in a short introductory video, featuring some of our researchers and patient advisors. The video was created and released on World Ovarian Cancer Day, May 8th 2023 through social media channels and has been viewed more than 300 times (available at: https://www.youtube.com/watch?v=llxtDPFwJts). We also translated the video into Bengali, with a view to encouraging members of the Bangladeshi community to get involved with PPI (Available at: https://www.youtube.com/watch?v=P3S8zrGAJ2g).

## Conclusion

Although it sounds obvious, simply talking to patients gives researchers a better perspective and insight into what is important to cancer patients and what improvements and developments they want to see. By combining our efforts, we can work towards solutions to the problems patients face every day.

## Data Availability

Not applicable.

## Abbreviations

PPI: Public and Patient Involvement
PARPi: PARP inhibitor
